# Correlation Between Glycated Hemoglobin (HbA1c) Levels and Lipid Profile in Patients With Type 2 Diabetes Mellitus at a Tertiary Hospital in Saudi Arabia

**DOI:** 10.7759/cureus.80736

**Published:** 2025-03-17

**Authors:** Ibrahim AlZeer, Aseel M AlBassam, Abdulaziz AlFeraih, Asma AlMutairi, Bayan AlAskar, Deema Aljasser, Fatimah AlRashed, Nawaf Alotaibi, Sara AlGhamdi, Zahra AlRashed

**Affiliations:** 1 Family Medicine, King Fahad Medical City, Riyadh, SAU; 2 Medicine, Dar Al Uloom University, Riyadh, SAU; 3 Family Medicine, Dar Al Uloom University, Riyadh, SAU

**Keywords:** dyslipidemia, glycemic control, hba1c, lipid profile, triglycerides, type 2 diabetes mellitus

## Abstract

Background: Type 2 diabetes mellitus (T2DM) is often associated with dyslipidemia, which increases the risk of cardiovascular disease. This study aimed to evaluate the relationship between glycated hemoglobin (HbA1c) levels and lipid profiles among newly diagnosed T2DM patients in Saudi Arabia.

Methods: A cross-sectional retrospective study was conducted at King Fahad Medical City in Riyadh between January 2022 and December 2023. Data on demographic factors, body mass index (BMI), blood glucose levels, and lipid profiles were analyzed.

Results: A total of 483 patients were included in the study, with the majority (42.2%) aged between 55 and 64 years. The study population consisted of 64.2% females with a mean BMI of 30.31±5.41. High HbA1c levels were observed in 68.3% of patients. Significant associations were found between HbA1c levels and triglycerides (TG) (p<0.001) as well as high-density lipoprotein cholesterol (HDL-c) (p<0.001), whereas low-density lipoprotein cholesterol (LDL-c) and total cholesterol (TC) showed no significant differences. Similarly, fasting blood glucose levels were associated with TG (p<0.001) and HDL-c (p=0.03) but not with TC or LDL-c. Regression analysis revealed that HDL-c was negatively correlated with HbA1c, whereas TG was positively correlated with HbA1c. No other parameter showed a significant correlation.

Conclusion: Poor glycemic control, as indicated by elevated HbA1c levels, is significantly associated with adverse lipid profile components, particularly higher TG levels and lower HDL-c.

## Introduction

When it comes to non-communicable disease burden, diabetes mellitus (DM) is one of the leading causes of death and disability worldwide and ranks among the fastest-growing health challenges worldwide [1]. In some countries, it is now reaching epidemic proportions. According to the World Health Organization (WHO), the number of people affected with DM has risen tremendously over the years, with 830 million affected in 2022 compared to 200 million in 1990 [2]. Saudi Arabia has witnessed a considerable rise in the prevalence of DM over the years, primarily due to a sedentary lifestyle. In Saudi Arabia, the prevalence of DM increased to more than 20% in recent years from 3.4% in 1996 [3]. DM is also related to a significant risk of complications that exaggerate the overall morbidity associated with the disease. Among various complications, dyslipidemia, hypertension, and atherosclerosis are quite common [4]. Diabetic patients are at high risk of developing cardiovascular diseases (CVD), and dyslipidemia can further increase this risk in individuals with type 2 diabetes mellitus (T2DM) [5]. Diabetic dyslipidemia refers to decreased concentration of high-density lipoprotein (HDL), elevated low-density lipoprotein cholesterol (LDL-c), total cholesterol (TC), and triglyceride (TG) levels [6].

According to the American Diabetes Association (ADA), diabetes can be diagnosed based on glycated hemoglobin (HbA1c) level of ≥6.5% (48 mmol/mol), while patients with prediabetes are defined by an HbA1c level between 5.7% and 6.4% (39-47 mmol/mol) [7]. HbA1c indicates the plasma glucose levels over the period of two to three months [8]. However, some factors can influence hemoglobin glycation in a way that is independent of glycemia levels in the body. These can include sickle cell anemia, erythropoietin therapy, hemodialysis, and loss of blood [7]. Despite these limitations, reasons supporting the use of HbA1c level in the diagnosis and monitoring of DM include no need for fasting, greater preanalytical stability, and a long-term assessment of glucose control [7]. HbA1c can not only help determine glycemic control but also help predict the risk of diabetes-related complications and even the risk of mortality [9,10].

In literature, several studies have evaluated lipid profile parameters in T2DM patients and their association with HbA1c levels. However, the evidence is conflicting as some studies have found a positive correlation between dyslipidemia and elevated HbA1c levels [3,11,12]. However, other studies did not find a significant correlation between these two parameters [13,14]. Therefore, further research is needed to confirm the findings of the previous studies. This study was carried out to find out if any association exists between HbA1c and the lipid profile of T2DM patients.

## Materials and methods

Study design and setting 

This cross-sectional study was conducted at King Fahad Medical City (KFMC) in Riyadh, Saudi Arabia. Data was obtained from the medical records of adult patients newly diagnosed with T2DM who attended the family medicine clinic at KFMC. The data was collected by a random sampling technique between January 1, 2022, and December 31, 2023.

Study population 

The inclusion criteria for the study were adult patients aged 45 years and older and patients newly diagnosed with T2DM. For the current study, the ADA criteria were used for diagnosis [15]. Patients with liver or renal impairment, CVD, and endocrine conditions were excluded due to the possibility of bias due to metabolism changes and medication use, which affect lipid profiles. Furthermore, patients using lipid-lowering medications were also excluded. Those patients who lacked complete files were also excluded from the present study.

Study variables 

The study included independent, dependent, and confounding variables. The primary independent variable is HbA1c levels, which influence the dependent variables, including lipid profile parameters such as TC, HDL-c, LDL-c, and TG. Additional variables included demographic data (age and gender) and body mass index (BMI), which will be treated as confounding factors depending on data availability.

Sample size

The sample size was calculated using OpenEpi version 3, an online epidemiological statistics tool assuming a large population size as the exact number of T2DM patients attending the healthcare centers within the timeframe may be unknown. For the calculation, a prevalence of 50% was assumed and a confidence level of 95% with a margin of error of 5% was considered. Based on these, the appropriate sample size was approximately 400 patients [3,16]. The formula used to calculate the sample size is n=(Z²×p×(1-p))/d². By substituting the values, we get n=(1.96²×0.5×0.5)/0.05², which results in n≈384.

Data analysis

The data analysis was performed by the Statistical Package of Social Science SPSS, version 27 (IBM Corp., Armonk, NY). Descriptive statistics were used to summarize patient demographics and clinical characteristics. Regression analyses were conducted to evaluate the associations between glycemic control and lipid parameters. Specifically, HbA1c levels were treated as the primary independent variable, while the lipid profile components TC, HDL-c, LDL-c, and TG were considered the dependent variables. In additional models, fasting blood glucose levels were also used as an independent variable to assess their relationship with the same lipid parameters. These analyses accounted for potential confounding factors, including age, gender, BMI, and marital status. A p-level of less than 0.05 was considered significant.

Ethical considerations 

Ethical approval was obtained from the King Fahad Medical City Institutional Review Board with numbers H-01-R-012, IRB00010471, and FWA00018774 prior to the study. Patient confidentiality and privacy were maintained according to the Helsinki Declaration. Patients' information was de-anonymized.

## Results

Sociodemographic details of the patients

A total of 483 patients were included in the study, with a majority being female (64.2%) compared to males (35.8%). The ages of participants ranged across four groups: 45-54 years (25.3%), 55-64 years (42.2%), 65-74 years (23.2%), and 75 years and above (9.3%). Most participants were non-smokers (95.2%), with only 4.8% identified as smokers. Regarding marital status, 71.4% were married, while 28.6% were single, divorced, or widowed (Table 1)

**Table 1:** Sociodemographic details of the patients included in the study

Characteristics	Frequency	Percentage (%)
Gender		
Female	310	64.2
Male	173	35.8
Age		
45-54 years	122	25.3
55-64 years	204	42.2
65-74 years	112	23.2
75 years and above	45	9.3
Smoking		
Smoker	23	4.8
Non-smoker	460	95.2
Marital status		
Single/divorced/widowed	138	28.6
Married	345	71.4

Clinical and biochemical parameters

Table 2 shows the clinical and biochemical parameters of the patients. BMI classifications showed that the majority of individuals were categorized as overweight (34%) or obese grade I (34.2%), with a mean BMI of 30.31±5.41, ranging from 17.47 to 57.45. Fasting blood glucose levels revealed that 52.1% had normal glucose levels, while 47.86% were high, with a mean value of 7.62±2.74 (range: 2.9-24.5). Regarding HbA1c levels, 31.7% had normal levels, while 68.3% exhibited high values, with a mean HbA1c of 7.28±1.41 (range: 4.45-12.52). Total cholesterol was normal in 84.3% of participants, and 15.7% had high levels, with a mean value of 4.27±1.11 (range: 0.66-8.62). For HDL-c, 69.6% of participants had normal levels, whereas 30.4% had low levels, with a mean HDL-c of 1.18±0.35 (range: 0.33-4.46). LDL-c levels were within normal limits for 55.9% of individuals, while 44.1% showed elevated levels, with a mean LDL-c of 2.65±1.01 (range: 0.84-6.82). Very-low-density lipoprotein (VLDL) levels were predominantly high, with 99.8% exceeding the normal threshold and a mean value of 3.14±0.99 (range: 0.6-7.6). Lastly, triglyceride levels were normal in 72.7% of participants, while 27.3% had high levels, with a mean triglyceride concentration of 1.45±0.8 (range: 0.38-6.24).

**Table 2:** Clinical and biochemical parameters of the patients BMI: body Mass Index; LDL: low-density lipoprotein; HDL: high-density lipoprotein; VLDL: very-low-density lipoprotein; TG: triglycerides

Parameters	N (%)	Minimum	Maximum	Mean	SD
BMI					
Underweight	3 (0.6)	17.47	57.45	30.31	5.41
Normal	71 (14.7)				
Overweight	164 (34)				
Obese grade I	165 (34.2)				
Obese grade II	57 (11.8)				
Obese grade III	23 (4.8)				
Fasting blood glucose					
Normal	244 (52.1)	2.9	24.5	7.62	2.74
High	224 (47.86)				
(Normal: Less than 7)					
HbA1c					
Normal	153 (31.7)	4.45	12.52	7.28	1.41
High	330 (68.3)				
(Normal: Less than 6.5%)					
Total cholesterol					
Normal	407 (84.3)	0.66	8.62	4.27	1.11
High	76 (15.7)				
(Normal: Less than 5.17)					
HDL-c					
Normal	336 (69.6)	0.33	4.46	1.18	0.35
Low	147 (30.4)				
(Normal: more than 1 mmol)					
LDL-c					
Normal	270 (55.9)	0.84	6.82	2.65	1.01
High	213 (44.1)				
(Normal: Less than 2.6)					
VLDL					
Normal	1 (0.2)	0.6	7.6	3.14	0.99
High	482 (99.8)				
(Normal: Less than 0.77)					
TG					
Normal	351 (72.7)	0.38	6.24	1.45	0.8
High	132 (27.3)				
(Normal: Less than 1.7)					

Relationship between HbA1c levels and lipid parameters

Table 3 shows the relationship between HbA1c levels and various lipid parameters. For HDL-c, a significant difference was observed between normal (mean: 1.2±0.3) and low levels (mean: 1.15±0.36), yielding a t-statistic of -3.5 and a p-value of <0.001. Similarly, TG exhibited a significant difference, with normal levels having a mean of 1.27±0.72 compared to 1.54±0.81 in high levels, reflected by a t-statistic of -5.5 and a p-value of <0.001. On the other hand, TC, LDL-c, and VLDL did not yield significant differences between the groups.

**Table 3:** Relationship between HbA1c levels and different lipid parameters LDL: low-density lipoprotein; HDL: high-density lipoprotein; VLDL: very-low-density lipoprotein; TG: triglycerides; TC: total cholesterol; HbA1c: glycated hemoglobin

Lipid parameters	HbA1c	Mean	Std. deviation	t-statistics	P-value
TC	Normal	4.21	1.09	-0.02	0.98
	High	4.3	1.1		
HDL-c	Normal	1.2	0.3	-3.5	<0.001
	High	1.15	0.36		
LDL-c	Normal	2.56	0.9	-0.88	0.38
	High	2.69	1.05		
VLDL	Normal	3.01	0.9	0.681	0.5
	High	3.2	1.03		
TG	Normal	1.27	0.72	-5.5	<0.001
	High	1.54	0.81		

Relationship between fasting blood glucose level and lipid parameters

Table 4 shows the association between fasting blood glucose levels and lipid parameters. For HDL-c, a significant difference was observed, with normal levels having a mean of 1.21±0.33 compared to 1.16±0.37 in high levels, yielding a t-statistic of -2.12 and a p-value of 0.03. Similarly, TG demonstrated a significant difference, with normal levels having a mean of 1.34±0.71 compared to 1.59±0.86 in high levels, reflected by a t-statistic of -3.5 and a p-value of <0.001. TC, LDL-c, and VLDL did not show a significant difference between normal and high fasting blood glucose levels.

**Table 4:** Relationship between fasting blood glucose level and different lipid parameters LDL: low-density lipoprotein; HDL: high-density lipoprotein; VLDL: very-low-density lipoprotein; TG: triglycerides; TC: total cholesterol

Lipid parameters	Fasting blood glucose	Mean	Std. deviation	t-statistics	P-value
TC	Normal	4.26	1.05	-0.85	0.4
	High	4.29	1.16		
HDL-c	Normal	1.21	0.33	-2.12	0.03
	High	1.16	0.37		
LDL-C	Normal	2.6	0.9	-0.70	0.48
	High	2.7	1.09		
VLDL	Normal	3.07	0.94	-0.95	0.34
	High	3.21	1.06		
TG	Normal	1.34	0.71	-3.5	<0.001
	High	1.59	0.86		

Associated determinants/factors affecting different lipid parameters

Age has a negative association with TC, with a beta coefficient of -0.137 and a significant p-value of 0.020. Other parameters showed correlation but did not achieve statistical significance (model adjusted R2=0.018, p<0.05) (Table 5). Age has a negative and statistically significant relationship with HDL-c (beta=-0.037, p=0.034) whereas gender has a strong positive association with significance with HDL-c (beta=0.186, p<0.001). HbA1c levels are negatively associated and significant (Beta=0.079, p=0.040), while BMI and fasting blood glucose levels do not show significant effects, with p-values of 0.912 and 0.763, respectively. Marital status has a negative and significant relationship (beta=-0.084, p=0.017) (model adjusted R2=0.10, p<0.001) (Table 5).

**Table 5:** Associated determinants/factors affecting different lipid parameters in patients BMI: body Mass Index; LDL: low-density lipoprotein; HDL: high-density lipoprotein; TG: triglycerides; TC: total cholesterol; HbA1c: glycated hemoglobin

Independent variable	Beta coefficient		Robust standard error	T values	P-values	Model unadjusted R2; adjusted R2; p-value
TC:						
Age		-0.137	-0.112	-2.340	0.020	0.032; 0.018; 0.029
Gender		0.200	0.086	1.695	0.091	
Smoking status		0.228	0.044	0.940	0.348	
HbA1c levels		0.161	0.067	1.264	0.207	
BMI		-0.099	-0.093	-1.946	0.052	
Fasting blood glucose levels		-0.024	-0.011	-0.202	0.840	
Marital status		0.039	0.016	0.341	0.734	
HDL-C:						
Age		-0.037	-0.097	-2.128	0.034	0.11; 0.10; <0.001
Gender		0.186	0.253	5.230	0.000	
Smoking status		-0.083	-0.050	-1.129	0.260	
HbA1c levels		-0.079	-0.104	-2.058	0.040	
BMI		0.002	0.005	0.111	0.912	
Fasting blood glucose levels		-0.011	-0.015	-0.301	0.763	
Marital status		-0.084	-0.108	-2.404	0.017	
LDL-C						
Age		-0.128	-0.115	-2.414	0.016	0.03; 0.01; 0.03
Gender		0.058	0.027	0.538	0.591	
Smoking status		-0.263	-0.055	-1.195	0.233	
HbA1c levels		0.168	0.077	1.449	0.148	
BMI		-0.067	-0.069	-1.443	0.150	
Fasting blood glucose levels		0.045	0.022	0.418	0.676	
Marital status		0.128	0.057	1.220	0.223	
TG:						
Age		-0.056	-0.064	-1.341	0.181	0.04; 0.03; 0.002
Gender		-0.175	-0.105	-2.088	0.037	
Smoking status		-0.081	-0.022	-0.472	0.637	
HbA1c levels		0.192	0.112	2.118	0.035	
BMI		0.005	0.006	0.124	0.901	
Fasting blood glucose levels		0.163	0.101	1.950	0.052	
Marital status		-0.025	-0.014	-0.299	0.765	

The regression analysis demonstrates that age has a negative and statistically significant association with LDL-c (beta=-0.128, p=0.016). Gender, smoking status, HbA1c levels, BMI, fasting blood glucose levels, and marital status show no significant relationships (model adjusted R2=0.01, p<0.05) (Table 5). The regression analysis reveals that gender has a significant negative association with TG levels (beta=-0.175, p=0.037), while HbA1c levels have a significant positive relationship (beta=0.192, p=0.035). Smoking status, BMI, fasting blood glucose levels, and marital status do not display significant associations (model adjusted R2=0.03, p<0.05) (Table 5).

## Discussion

The findings of the present study revealed significant associations between HbA1c levels and fasting blood glucose levels and some lipid parameters. For example, a significant inverse relationship between HbA1c and HDL-c was observed, with participants having higher HbA1c levels demonstrating lower HDL-c. This contrasts several studies from the literature that reported no significant association between HDL-c and HbA1c levels. Sharahili et al. did not report any significant association between HDL-c and HbA1c [3]. Similarly, another study from Saudi Arabia did not find a significant correlation between the two parameters [16,17]. However, several studies have also reported findings that are in line with the present study [18]. For example, Samdani et al. observed a significant negative correlation between HDL-c and HbA1c, similar to present results [17]. Interestingly, some studies have also reported a positive correlation between HDL-c and HbA1c [19].

Similarly, in the present study, a positive association was noted between HbA1c and TG, with elevated HbA1c correlating with higher triglyceride levels (p<0.001). Several previous studies have also reported similar results. Alzahrani et al. reported a significant positive relationship between TG and HbA1c in T2DM patients in Saudi Arabia [16]. Similarly, Hussain et al. and Ozder in their respective studies from Afghanistan and Turkey found a positive association between TG and HbA1c in T2DM patients [20,21]. However, Sarkar and Meshram did not report a significant association between Hb1Ac and TG [18]. Insulin resistance is primarily implicated in dyslipidemia in T2DM patients. Elevated TG levels in T2DM result from impaired insulin secretion or function, which increases hepatic VLDL production and delays the clearance of TG-rich lipoproteins due to higher substrate availability for TG synthesis [22].

TG/HDL-c ratio serves as a key marker of atherogenicity, endothelial dysfunction, and insulin resistance. Elevated TG/HDL-c levels are strongly linked to poor glycemic control, as patients with lower HbA1c levels tend to have significantly lower ratios [23]. In the present study, no significant association was found between LDL-c and HbA1c. This is consistent with previous studies published in Saudi Arabia [3,16]. Similarly, Sarkar et al., in their study, also reported similar results for the association between HbA1c and LDL-c [18]. However, several other studies found a significant positive correlation between HbA1c levels and LDL-c [20,24,25]. For instance, Hussain et al. reported that HbA1c was significantly associated with LDL-c levels, but the relationship with HDL-c was nonsignificant, which contradicts the findings of the current study [20]. 

The study consisted predominantly of females (64.2%), with most participants aged between 55 and 64 years (42.2%). This age group aligns with global data showing that the incidence of T2DM increases with age due to factors like reduced insulin sensitivity and age-related physiological changes [26]. Furthermore, the results of the study also showed that almost half of the participants were obese, whereas one-third were overweight. This aligns with previous evidence from the literature, which has clearly highlighted a higher prevalence of obesity in T2DM. Daousi et al., in their study, reported that 52% of patients diagnosed with T2DM were obese, and 86% were either obese or overweight [27]. A similar link between obesity and T2DM has been established in the literature [28,29].

In the present study, 27.3% of the participants had high levels of TG. This coincides with literature as Firdous and Khan reported that 38% of T2DM patients had elevated levels of TG [30]. In the present study, a cut-off point of 7% was used for HbA1c for differentiation of normal and high glycemic levels. Studies have shown that HbA1C of less than 7% can reduce CVD complications [31,32]. A study found that the complexity of CVD tends to increase with advancing age, elevated HbA1c levels, high LDL-c, increased TG, and reduced HDL-c levels [33]. Some authors also think that HbA1c serves not only as a reliable indicator of glycemic control but also as a predictor of dyslipidemia. A 1% rise in HbA1c above normal corresponds to an approximate increase of 35 mg/dL in average blood glucose levels. Notably, reducing HbA1c by 1% can lower the risk of microvascular complications by 40% [34].

The high prevalence of dyslipidemia observed in the present study and its association with T2DM in the Saudi population highlights several challenges and offers potential solutions for the future. In the present study, 15.7% of the patients had high levels of TC. Globally the prevalence of hypercholesterolemia is very high. Eastern Mediterranean has the third highest prevalence of hypercholesterolemia in the world, with 38.4% of adults having elevated levels of cholesterol [35,36]. Compared to the present study, Sharahili et al. reported a high prevalence of hypercholesterolemia in T2DM patients in their study [3]. Given the high prevalence of dyslipidemia in Saudi Arabia, even among non-diabetics, it is important that clinicians guide the patients regarding its management, especially T2DM patients. It is advised that diabetic patients should exercise regularly, with 30 minutes of mild-intensity exercise approximately four to six times a week [36]. It is also important that physicians should understand the correlation between T2DM and dyslipidemia.

There are several limitations to this study that should be considered when interpreting the results. The patients were being treated with various medications; however, those results were not analyzed due to the main focus on the relationship between HbA1c and lipid parameters. Secondly, the retrospective nature of the study did not allow accounting for dietary habits and lifestyle choices at the time of study, which could have influenced the results. As this is just a single-center study, generalizability to broader populations might not be possible. Future research should explore longitudinal trends in glycemic and lipid control, considering the impact of antidiabetic and lipid-lowering medications.

## Conclusions

In conclusion, this study showed that HbA1c has a significant positive correlation with TG levels and HbA1c, whereas a negative correlation was observed between HDL-c and HbA1c. However, some parameters, like LDL-c and TC levels, showed no significant difference. Similarly, fasting blood glucose levels were associated with TG and HDL-c. No other parameters showed a significant correlation. Some of the findings of the current study align with previous studies, whereas others are inconsistent. The study also identified that T2DM patients have a significantly higher prevalence of dyslipidemia. This study showed that poor glycemic control is associated with adverse lipid profile components. However, further research is needed to validate these findings in larger, prospective studies.
